# Arbuscular Mycorrhizas Reduce Nitrogen Loss via Leaching

**DOI:** 10.1371/journal.pone.0029825

**Published:** 2012-01-10

**Authors:** Hamid R. Asghari, Timothy R. Cavagnaro

**Affiliations:** 1 Faculty of Agriculture, Shahrood University of Technology, Shahrood, Iran; 2 School of Biological Sciences, Monash University, Clayton, Victoria, Australia; 3 Australian Centre for Biodiversity, Monash University, Clayton, Victoria, Australia; University of Tartu, Estonia

## Abstract

The capacity of mycorrhizal and non-mycorrhizal root systems to reduce nitrate (NO_3_^−^) and ammonium (NH_4_^+^) loss from soils via leaching was investigated in a microcosm-based study. A mycorrhiza defective tomato mutant and its mycorrhizal wildtype progenitor were used in this experiment in order to avoid the indirect effects of establishing non-mycorrhizal control treatments on soil nitrogen cycling and the wider soil biota. Mycorrhizal root systems dramatically reduced nitrate loss (almost 40 times less) via leaching, compared to their non-mycorrhizal counterparts, following a pulse application of ammonium nitrate to experimental microcosms. The capacity of AM to reduce nutrient loss via leaching has received relatively little attention, but as demonstrated here, can be significant. Taken together, these data highlight the need to consider the potential benefits of AM beyond improvements in plant nutrition alone.

## Introduction

Agricultural systems are being intensified to meet the world's increasing demand for food and fiber [1]. To meet these demands fertilizer use is expected to increase dramatically [2]. However, excess application and inefficient use of fertilizers can have considerable negative economic and environmental consequences. For example, nitrate (NO_3_^−^), a highly mobile form of nitrogen, is readily lost from agricultural lands via leaching [3]. This can lead to contamination of drinking water supplies and eutrophication of water bodies [4], [5], [6]. Thus, interception of nitrate before it leaches below the root zone of plants is a high priority, both in terms of improving fertilizer use efficiency, and reducing the risk of environmental degradation [7].

Most terrestrial plant species, including the majority of crops, form arbuscular mycorrhizas (AM) [8]. These associations, between plant roots and a specialized group of soil fungi, play an important role in plant acquisition of nutrients, including P, N, Zn and others [9], [10]. While most research has focused on P, there is increasing evidence of an important role for AM in acquisition of N from both inorganic [11], [12], [13], [14] and organic [15], [16] sources in the soil. Whereas AM are typically considered in terms of their potential to improve plant nutrition, they have also been found to have an important, but often overlooked, role to play in reducing the loss of nutrients (both P and N) via leaching [17], [18], [19]. Thus, maintaining and enhancing levels of AM in ecosystems where the risk of nutrient leaching is high may be important.

To study mycorrhizal functioning, plants that are colonized by arbuscular mycorrhizal fungi (AMF) are normally compared to plants that are not colonized by AMF. These non-mycorrhizal treatments are typically established by sterilizing soil to eliminate the fungi. However, soil sterilization changes soil chemistry, and eliminates other soil microbes involved in nutrient cycling [20].One option to overcome this issue is the use of mycorrhiza defective plant mutants, and their mycorrhizal wildtype progenitors, as a means of establishing non-mycorrhizal controls [13], [20], [21]. The advantage of this approach is that it allows for the direct investigation of mycorrhizal effects on soil and plant processes with the wider soil biota intact. This is particularly important with respect to the role of AM in increasing plant nitrogen acquisition, as the cycling of nitrogenin the soil, which is extremely rapid and dynamic, is in large part driven by microbial processes [7].

Here we present results of a microcosm-based study investigating the capacity of mycorrhizal and non-mycorrhizal root systems to reduce nitrate (NO_3_^−^) and ammonium (NH_4_^+^) loss from soils via leaching. A mycorrhiza defective tomato mutant, and its mycorrhizal wild-type progenitor [22] were used to establish mycorrhizal and non-mycorrhizal treatments. The results of this study are considered in the context of the potential for mycorrhizal root systems to reduce nutrient loss (especially nitrate) from soils via leaching.

## Materials and Methods

### Experimental design

A glasshouse experiment was carried out to investigate the effects of forming AM on the capacity of root systems to reduce soil nitrate and ammonium leaching. A mycorrhiza defective tomato mutant with reduced mycorrhizal colonization (named *rmc*) and its mycorrhizal wildtype progenitor (named 76R) [22] were used to establish mycorrhizal and non-mycorrhizal treatments in experimental microcosms, to which either ammonium nitrate or water (control) were added. This approach is that it avoids the need to sterilize soils to establish non-mycorrhizal controls [13], [20]. Thus, mycorrhizal and non-mycorrhizal plants can be compared with the wider soil biota, including those involved in soil N cycling, in-tact [23]; this is a key point of novelty of this experiment. Importantly, the growth of these genotypes is match when grown under a wide range of experimental conditions [13], [24], [25], [26],with one exception [27]. Furthermore, when grown in the absence of AMF the growth of the genotypes is matched [24], indicating that the mutation affecting the formation of AM by the *rmc* genotype has no pleiotropic effects on other plant processes.

### Soil, plants and nutrient addition

Microcosms, as described previously [19], were established as follows: a 30 mm layer ofdried washed sand (140 g) was placed on a layer of cotton mesh at the base of PVC columns (90 mm diameter×400 mm deep) with a PVC cap (with a central hole, 15 mm in diameter) on their base. To each column 2.5 kg of a soil∶sand mixture (40∶60% W/W) was added to a final bulk density of 1.4 g.cm^−3^. A soil∶sand mix was used in this experiment as it provides a very even mixture, uniform leaching conditions, and ready extraction of roots and hyphae at the time of harvest [17], [19], [28]. The soil, which was air-dried and passed through a 2 mm sieve, was collected from the 0–15 cm layer of restored riparian zone adjacent to Faithfuls Creek in the southern Murray-Darling Basin in southeastern Australia (see www.mdba.gov.au/riparian-restoration-experiment/). The soil at this site is a quaternary red chromosol, fluvial silt-sand, with a pH of 6.0, plant available (Olsen) P of 4.5 mg kg^−1^, total C of 1.9% and total N of 0.12% (T.R. Cavagnaro, unpublished). This soil was selected as riparian zones commonly experience large nutrient inputs in rapid “pulse–based” events (as simulated here, see below), for example, following large rainfall events or at the break of seasons or droughts [29]. The sand used in the mix was a coarse grained and washed river sand, as in our earlier work on soil leaching [19].

Seeds of a mycorrhiza defective tomato (*Solanum lycopersicum* L. mutant (*rmc*), and its wildtype progenitor (76R) [22] were surface sterilized and imbibed prior to planting [19]. Seeds of either genotype were planted in each column, kept moist with RO water, and after 2 weeks, seedlings were thinned to one per column. Columns were then irrigated (to weight) with RO water every second day, to 80% of the field capacity, thereby ensuring that no water leached out of the columns during the plant growth phase of the experiment [17]. Plants were grown in a glasshouse with supplemental lighting: mean day time temperature was 22.1°C, min 18.3°C, max 25.9°C; night time temperature was mean 20.1°C, min 17.3°C, max, 22.6°C; and mean daily photon load of 495.1±108.6 mol quanta m^−2^. Four weeks after planting, all plants were supplied with a 20 ml of a modified Long Ashton nutrient solution minus P [30], once a week for 3 weeks. Each treatment was replicated four times; however, one replicate was lost from the *rmc* treatments during the course of the experiment.

### Nutrient addition treatments

Nine weeks after planting half of the pots were supplied with a pulse of nitrogen as 143 mg of ammonium nitrate (NH_4_NO_3_) dissolved in 10 ml RO water; this nitrogen addition treatment was equivalent to an input of 280 kg N ha^−1^ [31]. This amount of N, which was used in earlier leaching experiments using this soil [19], is within the range applied to commercial tomato crops. To the remaining control columns, 10 ml of RO water was added. Following the addition of the N pulse (or RO water for the controls), the cores were watered to 80% of field capacity, in order to water in the added nutrients, and to maintain constant soil moisture content for the remainder of the experiment.

### Harvesting and leachate collection

Ten weeks after planting (i.e. 7 days after nutrient addition treatments were applied) all cores were destructively harvested. The shoots of plants were removed (to eliminate water loss via transpiration), and the columns immediately flushed with 700 ml of RO water to leach soil nutrients from the columns. This approach was taken in an effort to simulate a large rainfall event as typically occurs at the site from where the soil used in this experiment was collected. The leachate was collected from the columns until the cessation of leaching (24 hrs) and the concentration of NH_4_^+^-N and NO_3_^−^-N determined colourimetrically, as for soil extracts (see below). Soil samples were collected from three layers (0–5, 10–15, 20–25 cm) for analysis of soil NO_3_^−^-N and NH_4_^+^-N concentrations (in duplicate). Briefly, the soils were extracted using a 2 M KCl solution and inorganic nitrogen content determined colorimetrically using a modification of the methods of Miranda et al. [32] for NO_3_^−^ (plus NO_2_) and Forster [33] for NH_4_^+^. Mycorrhizal hyphal length was determined [34] on the middle soil layer;preliminary analyses revealed that hyphal length densities did not differ between soil layers (data not shown). The roots were then carefully washed from all of the remaining soil with RO water. Mycorrhizal colonization of a sub-sample of roots was determined using the gridline intersect method [35], following clearing and staining of roots with Trypan Blue (omitting phenol from all reagents) [36]. All remaining plant material was dried at 60°C, and shoot dry weights (SDW) and root dry weights (RDW) determined. Plant material was then ground to a fine powder and the concentration of nitrogen determined by dry combustion (CHN 2000 analyzer, LECO Corporation, USA). Here we present plant nutrient data on a whole plant nutrient content basis (i.e. N per plant), rather than on a tissue concentration basis, as our emphasis is on nutrient interception, rather than nutrient concentrations in plant tissues (see results).

### Statistical analysis

Data from mycorrhizal treatments and nutrient addition treatments were analyzed using two-way analysis of variance (ANOVA) using JMP® (version 8.0.2. SAS Institute). Where significant differences between treatments were found (*P*<0.05), differences between individual treatment means were determined using Tukeys HSD tests. Additional targeted data analysis was undertaken to further explore genotypic differences (using t-tests) within the different nutrient addition treatments; these additional targeted analyses are indicated in the relevant sections of the results.

## Results

### Mycorrhizal colonization, plant growth and nutrition

Roots of the 76R genotype were well colonized, with levels of colonization not significantly different between the nitrogen (52±8% root length colonized: values are mean ± SE) and control (39±6% root length colonized) nutrient addition treatments. Conversely, colonization of *rmc* roots was less than 1%, and was restricted to the root epidermis. The length of external hyphae of arbuscular mycorrhizal fungi (AMF) was significantly higher in columns containing 76R plants (10.9±2.2 and 16.0±1.2 m.g^−1^ dry soil, for the nitrogen and control nutrient addition treatments, respectively) compared to those containing *rmc* plants (4.2±1.1 and 5.0±3.1 m.g^−1^ dry soil, for thenitrogenand control nutrient addition treatments, respectively). There was no significant difference in hyphal length densities between nutrient addition treatments.

**Figure 1:**
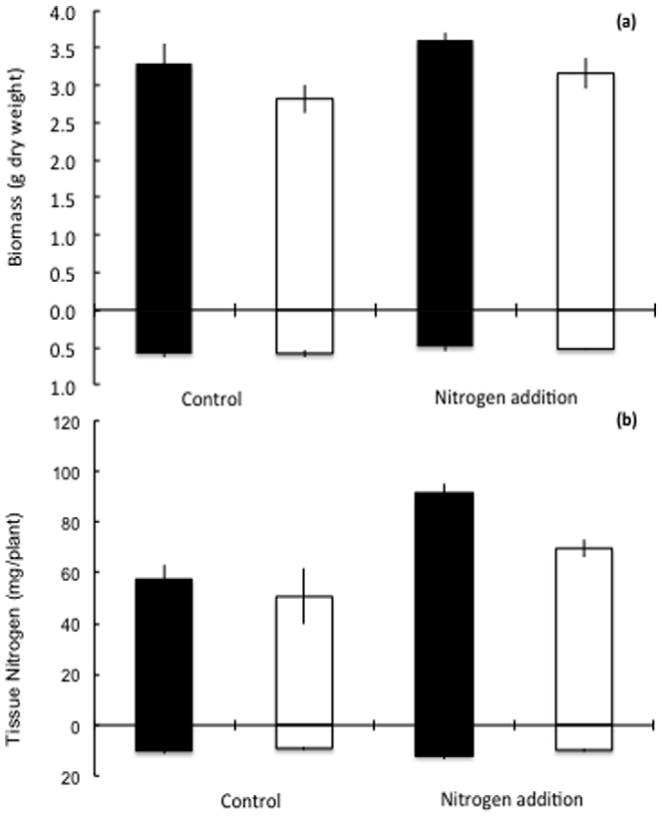
Mean shoot (above *X*-axis) and root (below *X*-axis). (a) dry weights, and (b) plant nitrogen contents, of the 76R (black bars) and *rmc* (white bars) genotypes of tomato, following the application of nutrient addition treatments (nitrogen or control). Values are mean ± S.E. The shoot dry weight of 76R plants was significantly greater than of *rmc* plants, irrespective of nutrient addition treatments; the nitrogen content differed significantly between genotypes irrespective of nutrient addition treatments, and vice versa; see text for details.

The RDW of plants did not differ between any of the treatments (Fig. 1a). Whereas, the SDW (Fig. 1a) of the 76R genotype was greater than that of the *rmc* genotype (F_(1,3)_ = 5.06, *P*<0.05), irrespective of nutrient addition treatment, total plant dry weight (SDW+RDW) did not differ between any of the treatments (see Fig. 1a). Shoot nitrogen content (mg.plant^−1^) of 76R plants was higher (F_(1,3)_ = 4.95, *P* = 0.05) than that of *rmc* plants (Fig. 1b).Similarly, the nitrogen content of plants grown in the nitrogen addition treatment (irrespective of genotype) was significantly (F_(1,3)_ = 17.32, *P* = 0.0019) higher than that of plants in the control (water addition) treatment. There were no significant differences in root nitrogen contents (Fig. 1b) between any of the experimental treatments. Importantly, whole plant nitrogen content (shoots+roots) was significantly higher in the 76R than *rmc* genotype, irrespective of nutrient addition treatment, and in the nitrogen addition treatment, irrespective of genotype (see Figure 1b).

### Soil nitrogen pools and interception

**Figure 2:**
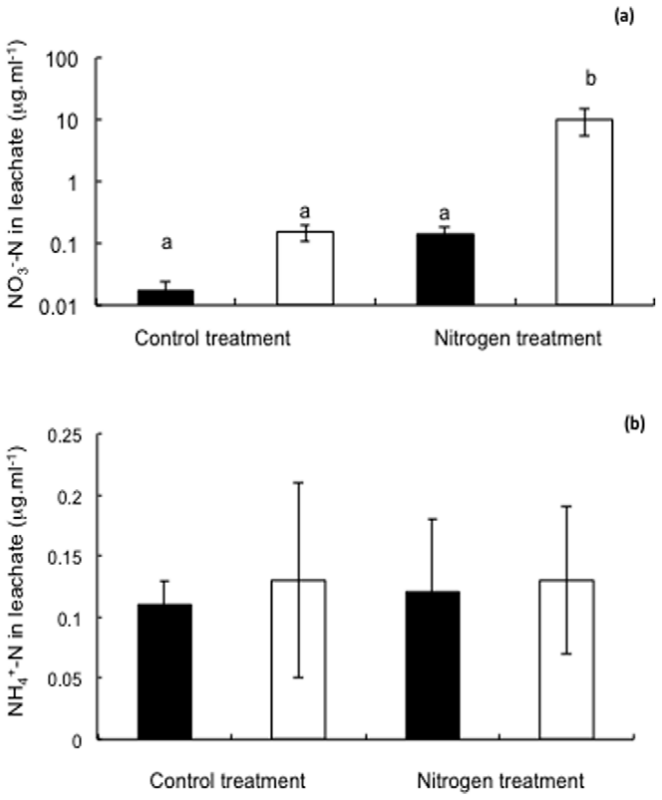
Concentration of (a) NO_3_^−^-N and (b) NH_4_^+^-N in leachate, collected from columns containing 76R (black bars) and *rmc* (white bars) genotypes of tomato, following the application of nutrient addition treatments (nitrogen or control). Values are mean ± S.E. Means followed by the same letter are not significantly different at the *P*<0.05 level. See text for results of targeted statistical analyses comparing genotypes within specific nutrient addition treatments.

The volume of leachate collected from the experimental columns did not differ between any of the experimental treatments (477 ml±48 ml per column). The concentration of nitrate in the leachate collected from columns (Fig. 2a) containing *rmc* root systems was significantly higher in the nitrogen addition treatment, when compared to all other treatments (Fig. 2a) (F_(1,3)_ = 160.75, *P*<0.0001).When the control treatment was considered separately from the nitrogen addition treatment (targeted t-test), the concentration of NO_3_^−^ leached from columns containing *rmc* plants was significantly higher than that from columns containing 76R plants. The concentration of ammonium in the leachate was low, and did not differ between any of the experimental treatments (Fig. 2b).

**Figure 3:**
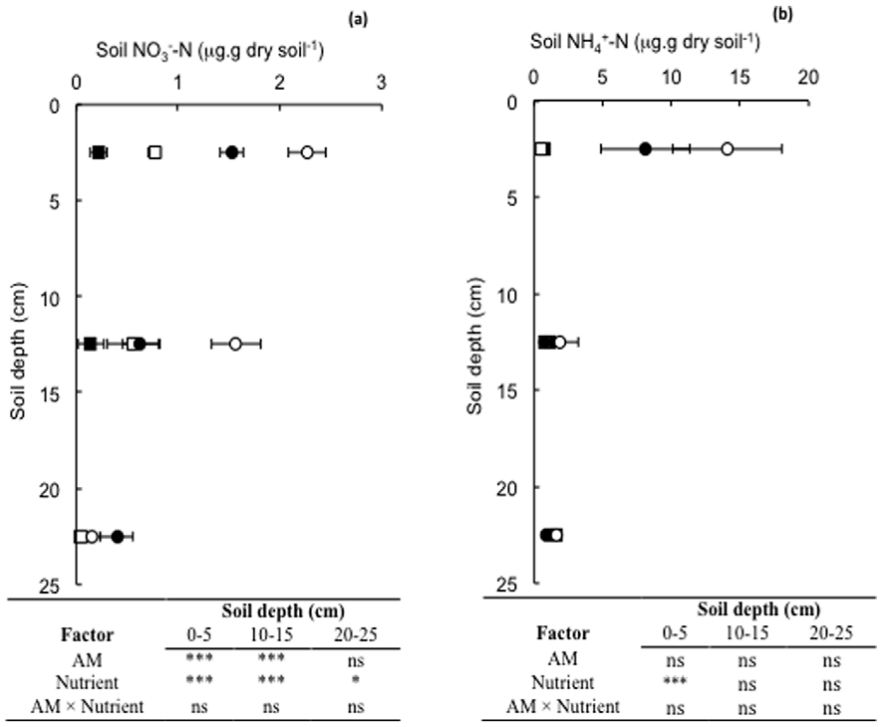
Soil (**a**) NO_3_^−^-N and (**b**) NH_4_^+^-N, concentrations with depth (data plotted at mid-point of sampling depth) in columns containing mycorrhizal (closed symbols) and non mycorrhizal (open symbols) tomato plants in the nitrogen addition (circular symbols) and water control nutrient addition (square symbols) treatments. Values are means ± standard error. ANOVA Tables are given below Figures, see text for additional details of statistical analysis. *, **, *** = significant at *P*<0.05, 0.01, 0.001 levels respectively. ns = not significant, *P*>0.05.

At the end of the experiment, the amount of N-NO_3_^−^ remaining in the soil was relatively low (Fig. 3a); the concentration of N-NO_3_^−^ was higher in the nitrogen addition treatment, in the surface (and to a lesser extent the middle and lower) soil layer, irrespective of genotype. Similarly, the concentration of N-NO_3_^−^ was lower in the surface and middle soil layers, where plants were mycorrhizal, irrespective of nutrient addition treatment. The concentration of N-NH_4_^+^ in the soil (Fig. 3b) at the end of the experiment was higher in the surface soil layers, than at depth. Ammonium was especially high in the nitrogen addition treatment, irrespective of genotype.

## Discussion

Mycorrhizal root systems dramatically reduced nitrate loss (almost 40 times less) via leaching, compared to their non-mycorrhizal counterparts, following a pulse application of ammonium nitrate to experimental microcosms. The capacity of AM to reduce nutrient loss (both N and P) via leaching has received relatively little attention, but is potentially very significant [17], [18], [19]. A decrease in nitrate loss via leaching on the scale seen here may be especially important in ecosystems where the risk of leaching is high, such as at the interface of agricultural and natural lands [4], [5], [6]. Taken together, these data highlight the need to consider the potential benefits of AM beyond improvements in plant nutrition alone.

The decrease in nitrate loss from columns containing mycorrhizal root systems seen here cannot solely be attributed to size asymmetry between mycorrhizal and non-mycorrhizal plants. Importantly, the mycorrhizal tomato genotype was well colonized, whereas the mycorrhiza defective tomato mutant was not colonized. By effectively increasing the absorptive surface of root system, AMF allow exploration of a larger soil volume, and hence, access to more nutrients [8]. The reduction in nitrate leachate from columns containing mycorrhizal plants was paralleled by a lower concentration of nitrate in the soil and higher plant nitrogen content. This is consistent with earlier work demonstrating that AM enhance the capacity of plants to acquire N from inorganic sources, under both laboratory and field conditions [11], [12], [13], [14], [37]. Further, using the same genotypes as in the present study Ruzicka et al. [14] found that the regulation of key genes involved in the N transport and assimilation indicating a shift towards N uptake via the mycorrhizal pathway in the mycorrhizal genotype. Together, this suggests that the reduction of nitrogen lost via leaching of nitrate was due to enhanced nitrogen interception and immobilization by mycorrhizal root systems [12], [38].

Given its relatively low mobility in soils, it is not surprising that there was little ammonium was lost via leaching [39]. Although most of the added ammonium was retained in the upper soil layer, mycorrhizal interception of ammonium can be important [12], which may indirectly reduce the risk of nitrogen loss as nitrate. That is, ammonium not taken up by roots or AMF can be readily transformed into nitrate under aerobic conditions (via nitrification), such as those in the columns during the week following the application of ammonium nitrate to the soil [40]. This example serves to highlight the need for carefully controlled studies of soil-plant-AMF-nitrogen dynamics.

The use of a mycorrhiza defective tomato mutant and its mycorrhizal wildtype progenitor to establish mycorrhizal treatments avoided the use of non-specific sterilization techniques to establish non-mycorrhizal controls, which can eliminate soil biota and alter soil nitrogen cycling [13], [20]. This allows us to attribute the differences seen here to the mycorrhizal status of the root systems. It may also explain why the reduction in N lost via leaching in the present study is substantially greater than in our earlier work using soil sterilization for the establishment of non-mycorrhizal controls [19]. While the very large differences in nitrate loss via leaching were likely due to plant/AMF immobilization, it will be important in future studies to begin to consider potential changes in microbial communities in the rhizosphere of these genotypes, as have been reported in earlier studies [27]. For example, possible differences in rhizodeposition between the genotypes, which may in turn affect denitrification and other nitrogen cycling processes, may need to be considered.

To further explore the role of AMF in reducing nitrate loss via leaching, the use of nitrogen isotopes will be especially informative. For example, the capacity to trace the applied nitrogen through the soil, plant, leachate and atmospheric (i.e. N_2_O and N_2_ efflux) poolswould permit calculation of full nitrogen loss budgets, and the impacts of AM on them. Similarly, studies with in-tact soil cores (rather than a soil-sand mix) and field based experiments are also likely to be especially informative. Nevertheless, it is clear from the data presented here, and in our earlier studies [19], that mycorrhizal root systems (i.e. roots plus fungi) can have a role to play in reducing nitrate loss via leaching.

### Conclusions

The importance of AM in improving plant nutrition has been long known [8]. The results of this study highlight the potential of AM to reduce nitrate leaching from soil on a previously unrecognized scale. This suggests that managing farming systems to maximize mycorrhizal colonization of roots, especially where the risk of nitrate loss via leaching is high, should be of high priority [7]. Similarly, maintaining and enhancing mycorrhizas in vegetated buffer strips between potential sources of nitrogen pollution, such as farms and urban areas, and potential sinks for nitrogen, such as natural lands and water bodies, is also important. Effective management of AM in such systems will have the benefits of reducing the risk of nitrogen loss via leaching, as well as improving plant nitrogen nutrition.
